# Mannose-binding lectin genotypes: lack of association with susceptibility to thoracic empyema

**DOI:** 10.1186/1471-2350-11-5

**Published:** 2010-01-15

**Authors:** Stephen J Chapman, Fredrik O Vannberg, Chiea C Khor, Anna Rautanen, Nicholas A Maskell, Christopher WH Davies, Catrin E Moore, Nicholas P Day, Derrick W Crook, Robert JO Davies, Adrian VS Hill

**Affiliations:** 1 The Wellcome Trust Centre for Human Genetics, University of Oxford, UK; 2 Oxford Centre for Respiratory Medicine, Oxford Radcliffe Hospitals, Oxford, UK; 3 Department of Respiratory Medicine, Royal Berkshire Hospital, Reading, UK; 4 Centre for Tropical Medicine, Nuffield Department of Clinical Medicine, Oxford Radcliffe Hospitals, Oxford, UK; 5 Department of Microbiology, John Radcliffe Hospital, Oxford, UK

## Abstract

**Background:**

The role of the innate immune protein mannose-binding lectin (MBL) in host defence against severe respiratory infection remains controversial. Thoracic empyema is a suppurative lung infection that arises as a major complication of pneumonia and is associated with a significant mortality. Although the pathogenesis of thoracic empyema is poorly understood, genetic susceptibility loci for this condition have recently been identified. The possible role of MBL genotypic deficiency in susceptibility to thoracic empyema has not previously been reported.

**Methods:**

To investigate this further we compared the frequencies of the six functional *MBL *polymorphisms in 170 European individuals with thoracic empyema and 225 healthy control individuals.

**Results:**

No overall association was observed between MBL genotypic deficiency and susceptibility to thoracic empyema (2 × 2 Chi square = 0.02, *P *= 0.87). Furthermore, no association was seen between MBL deficiency and susceptibility to the Gram-positive or pneumococcal empyema subgroups. MBL genotypic deficiency did not associate with progression to death or requirement for surgery.

**Conclusions:**

Our results suggest that MBL genotypic deficiency does not associate with susceptibility to thoracic empyema in humans.

## Background

Mannose-binding lectin (MBL) is a serum lectin which binds repeating sugar arrays on the surface of a wide range of micro-organisms, including Gram-positive bacteria [1]. MBL appears to play an important role in innate immunity by promoting opsonophagocytosis. This occurs primarily through the activation of complement independently of antibody after binding MBL-associated serine proteases (MASPs) 1 and 2. Significant inter-individual variation in baseline serum MBL levels and function occurs as a result of six well-described genetic polymorphisms in the *MBL2 *gene: three structural polymorphisms and three promoter polymorphisms [2]. The structural polymorphisms consist of non-synonymous polymorphisms in codons 52, 54, or 57, where the mutant alleles are individually referred to as D, B and C, respectively. A coding region containing any of the D, B or C mutations is referred to as O, and the wild-type allele at each locus is referred to as A. The structural mutations impair triple helix formation in the MBL collagenous tail, leading to degradation and functional deficiency of MBL. Serum MBL concentrations are 10-20% of that expected in heterozygotes (A/O) for the structural polymorphisms, and virtually undetectable in functional homozygotes (O/O; individuals who are homozygous or doubly heterozygous for two different coding mutations).

Polymorphisms in the promoter region of the *MBL2 *gene have also been shown to exert functional effects on MBL transcription. These polymorphisms are located at positions -550 (H/L) and -221 (Y/X), as well as at position +4 (P/Q) in the untranslated region of exon 1. Of these, the -221 polymorphism has the strongest effect on MBL serum concentration, with the Y allele associated with high and the X with low MBL expression. Indeed, individuals heterozygous for X and O have virtually undetectable serum levels of MBL. The number of haplotypes observed is limited as a result of linkage disequilibrium between the six polymorphisms; of particular note, X is observed only on chromosomes with wild-type structural alleles (A). The functional consequences of XA homozygosity remain controversial, although some studies have reported very low MBL concentrations and considered this to be a deficient state [2-4].

In contrast to the structural (A/O) and -221 (Y/X) polymorphisms, the functional effects of the L/H and P/Q polymorphisms appear to be relatively minor. Studies performed in transfected cell lines suggest that the haplotype variants HY, LY and LX demonstrate high, medium and low promoter activity, respectively [5]. Within the haplotype LY, the presence of a Q allele at position +4 appears to result in marginally higher serum MBL concentrations than observed with the P allele [5].

Not all of the MBL in serum is functional, and there is considerable debate as to whether measurement of serum concentration, functional activity or genotypes is the optimal method of assessing an individual's MBL status [4]. The majority of studies investigating disease associations have examined MBL genotypes. MBL genotypic deficiency has been reported in association with a wide range of infectious phenotypes, including susceptibility to generalised childhood infection, invasive pneumococcal disease, severe sepsis, meningococcal sepsis, severe infection following chemotherapy and allogeneic haemopoietic stem cell transplantation, and prolonged duration of neutropaenic fever in children with malignancy, in addition to susceptibility to autoimmune conditions such as systemic lupus erythematosus [3,4,6-13].

Previous studies have not however investigated the possible role of MBL deficiency in the development of thoracic empyema, a suppurative infection of the pleural cavity which arises as a complication of pneumonia and is associated with a mortality rate of approximately 15% [14]. Although the pathogenesis of thoracic empyema is poorly understood, genetic susceptibility loci for this condition have recently been identified in humans [15,16]. Given the well-described function of MBL in innate immunity, and its more controversial role in pneumonia pathogenesis, we hypothesised that MBL genotypic deficiency might associate with susceptibility to thoracic empyema. To investigate this further we studied the frequencies of the six functional *MBL2 *polymorphisms in individuals with thoracic empyema, as well as a control group.

## Methods

### Sample information

The thoracic empyema study group has previously been described [14,15]. Briefly, blood samples were collected from patients with thoracic empyema recruited upon entry to the UK MIST1 trial [14]. The diagnosis of thoracic empyema was defined by the presence of pleural fluid that was macroscopically purulent or positive on bacterial culture or Gram's stain, or by a pleural fluid with pH < 7.2 in the setting of clinical evidence of infection [14]. The mean age of individuals with empyema in this study was 60 years; 70% were male. The bacteriology of the cohort was described in detail: analysis of pleural fluid was performed using both standard bacterial culture and amplification and sequencing of the bacterial 16 s ribosomal RNA gene [17]. Using these techniques, a bacteriological diagnosis was made in 59% of the available samples, with the *Streptococcus intermedius-anginosus-constelatus (milleri) *group and *Streptococcus pneumoniae *being the dominant isolates.

The control group comprised 225 cord blood samples. Blood was collected anonymously from the discarded umbilical cords of healthy neonates born at the John Radcliffe Hospital, Oxford, UK, as previously described [15]; 54% of the cord blood donors were male. Examination of microsatellite markers excluded contamination with maternal DNA.

Individuals of non-European ancestry were excluded from cases and controls. The study was approved by the research ethics committees of the participating hospitals and informed consent was obtained from all participants.

### Genotyping techniques and analysis

DNA extraction from blood was performed using Nucleon II kits (Scotlab Bioscience, Buckingham, UK). All six functional *MBL2 *polymorphisms were studied in the thoracic empyema and control study groups. The three promoter polymorphisms were genotyped using the Sequenom Mass-Array^® ^MALDI-TOF primer extension assay system [18]. A touch-down PCR protocol was used, with cycling conditions as follows: 95°C for 15 minutes; 94°C for 20 seconds; 65°C for 30 seconds; 72°C for 30 seconds; steps 2 to 4 repeated for 5 cycles; 94°C for 20 seconds; 58°C for 30 seconds; 72°C for 30 seconds; steps 5 to 7 repeated for 5 cycles; 94°C for 20 seconds; 53°C for 30 seconds; 72°C for 30 seconds; steps 8 to 10 repeated for 38 cycles; final extension at 72°C for 3 minutes. Primer sequences are listed in table 1. Each genotyping plate contained a mixture of case and control samples. The three structural *MBL2 *polymorphisms were genotyped by direct sequencing, with all three polymorphisms visualised from a single sequencing reaction; primer sequences are listed in Table 1. General PCR conditions for amplifying products prior to sequencing were as follows: 95°C for 15 minutes, and then 40 cycles of 95°C for 30 seconds, 55-65°C for 30 seconds, and 72°C for 60 seconds, followed by 72°C for five minutes. Direct sequencing was performed using BigDye v3.1 terminator mix (ABI) followed by ethanol precipitation. Plates were run on an ABI 3700 capillary sequencer and sequence analysis was performed with the Lasergene DNAstar package (Lasergene), using SeqMan software. Statistical analysis of genotype associations and logistic regression was performed using the program SPSS v12.0.

**Table 1:** Primer sequences for *MBL2 *polymorphism genotyping

Polymorphism	Primer sequence
-550 L/H	Forward - ACGTTGGATGGAGAAAATGCTTACCCAGGC
	Reverse - ACGTTGGATGCAACCCAGCCCAGAATTAAC
	Extension - CTTACCCAGGCAAGCCTGT

-221 Y/X	Forward - ACGTTGGATGACGGTCCCATTTGTTCTCAC
	Reverse - ACGTTGGATGTTCATCTGTGCCTAGACACC
	Extension - CCCATTTGTTCTCACTGCCAC

+4 P/Q	Forward - ACGTTGGATGACCCAGATTGTAGGACAGAG
	Reverse - ACGTTGGATGGTGAGAAAACTCAGGGAAGG
	Extension - GTAGGACAGAGGGCATGCT

MBL2 structural polymorphism sequencing	Forward - GCACCCAGATTGTAGGACAGAG
	Reverse - CAGGCAGTTTCCTCTGGAAGG

## Results

None of the *MBL2 *polymorphisms were individually associated with susceptibility to empyema. In particular, the mutant allele frequencies of the three structural polymorphisms did not significantly differ between empyema cases and controls (D allele frequency 0.051 in cases and 0.07 in controls, 2 × 2 Chi square = 1.13, *P *= 0.29; B allele frequency 0.13 in cases and 0.145 in controls, 2 × 2 Chi square = 0.33, *P *= 0.57; C allele frequency 0.039 in cases and 0.02 in controls, 2 × 2 Chi square = 2.52, *P *= 0.11). As discussed above, the functional level of MBL is determined not however by individual polymorphisms but by the combinations of different alleles. In particular, the structural polymorphisms (A/O) and the -221 promoter polymorphism (X/Y) exert the strongest effects on MBL function, and the majority of studies have concentrated solely on these polymorphisms [5]. To summarise the earlier descriptions of the functional consequences of different MBL genotypes, individuals who are A/A (i.e. no MBL structural variant alleles) or YA/O (heterozygotes for a single structural variant allele, i.e. A/B, A/C or A/D) are functionally normal, whereas individuals who have the genotypes O/O (homozygotes for variant alleles B/B, C/C or D/D, or compound heterozygotes e.g. B/C) or XA/O (heterozygous for the X promoter allele and a structural variant allele) are functionally deficient. The functional consequences of XA homozygosity are controversial, although some studies have considered this to be a deficient state. Taking this approach, the results of MBL functional status in the thoracic empyema and control groups are presented in Table 2.

**Table 2:** MBLfunctional status (-221, 52, 54, and 57 polymorphisms) in individuals with thoracic empyema and controls.

MBL functional status	Genotypes	Controls (%)	Empyema (%)
MBL Deficient	O O	17 (7.6%)	13 (7.8%)
	
	XA O	18 (8.0%)	13 (7.8%)
	
	XA XA	16 (7.1%)	13 (7.8%)
	
	Total	51 (**22.7%**)	39 (**23.4%**)

MBL Sufficient	XA YA	53 (23.6%)	35 (21.0%)
	
	YA O	49 (21.8%)	33 (19.8%)
	
	YA YA	72 (32.0%)	60 (35.9%)
	
	Total	174 (**77.3%**)	128 (**76.6%**)

Overall total		225	167

No overall association was observed between MBL genotypic deficiency (defined on the basis of B, C, D, and X) and susceptibility to thoracic empyema (2 × 2 Chi square = 0.02, *P *= 0.87; Table 2). To acknowledge the controversy surrounding the functional consequences of XA homozygosity, the analysis was repeated with the XA/XA state considered as MBL sufficient; a lack of overall association with thoracic empyema susceptibility was again observed (*P *= 0.99). On subgroup analysis a lack of association was also noted between MBL deficiency and susceptibility to Gram-positive empyema (cases n = 71; *P *= 0.98) or pneumococcal empyema (cases n = 23; *P *= 0.92). The functional consequences of the L/H and P/Q polymorphisms are less well-defined; in any case, there was no apparent association with susceptibility to thoracic empyema (Table 3). Logistic regression analysis demonstrated no effect of age, gender or comorbid conditions on *MBL2 *genotype. Within the thoracic empyema group, 49 patients had either died (n = 26) or required surgery (n = 24) after 12 months; there was no apparent association between MBL genotypic deficiency and these adverse outcomes (*P *= 0.5).

**Table 3:** *MBL2 *L/H and P/Q polymorphisms in individuals with thoracic empyema and controls.

Polymorphism	Genotypes	Controls (%)	Empyema (%)
-550 (H/L)	CC	88 (45.1%)	75 (51.4%)
	
	CG	85 (43.6%)	52 (35.6%)
	
	GG	22 (11.3%)	19 (13.0%)
	
	3 × 2 Chi square	2.21	
	
	*P *value	0.33	

+4 (P/Q)	CC	116 (54.5%)	82 (52.2%)
	
	CT	85 (39.9%)	66 (42.0%)
	
	TT	12 (5.6%)	9 (5.7%)
	
	3 × 2 Chi square	0.19	
	
	*P *value	0.91	

## Discussion

As with other infectious diseases, the development of thoracic empyema in a particular individual is likely to reflect a combination of multiple acquired and genetic risk factors. Common genetic variants in the immune response genes *PTPN22 *and *NFKBIA *have previously been identified in association with empyema susceptibility [15,16], although additional genes are likely to be involved. Here we show that genotypic deficiency in MBL does not associate with susceptibility to thoracic empyema. The sample size is sufficient to detect an odds ratio of 2.0 for MBL deficient genotypes at a significance threshold of 0.05 with 80% power. The small size of the 'adverse outcomes' subgroup (death or need for surgery) however precludes a firm conclusion regarding the possible effect of MBL deficiency on empyema clinical outcome.

The genotype frequencies in the healthy neonates used as a control group in this study are broadly similar to those noted in adult studies of European individuals [19,20], and the control allele frequencies for each of the structural variants are near-identical to those described in European populations [4]. The frequency of O/O homozygotes was noted to be 7.6% in the neonatal controls, slightly higher than the frequencies of 5-6% noted in studies of apparently healthy European adults. Genotyping error is highly unlikely to account for this difference, as genotyping of these variants was performed by direct sequencing in our study. It is theoretically possible that MBL-deficient individuals are at significant risk from premature death and this accounts for an overall lower frequency of genotypic deficiency in adults; however such an effect has not been demonstrated in a large population-based study of MBL deficiency [20] and furthermore in our study we did not observe an effect of age on MBL status during regression analysis. This difference is therefore likely to simply reflect chance variation in population sampling.

The role of MBL deficiency in susceptibility to respiratory infection remains controversial. Early reports described an association between MBL deficiency and susceptibility to invasive pneumococcal disease [7,8], and more recently studies have investigated its possible role in susceptibility and outcome from pneumonia. A study of Spanish patients reported a lack of association between MBL genotypic deficiency and susceptibility to community-acquired pneumonia, although possible associations between deficiency and poor clinical outcomes were noted [21]. A second study has demonstrated a lack of association between MBL deficiency and both susceptibility to community-acquired pneumonia and outcome from this infection [19]. Another recent report described an increased risk of death associated with MBL deficiency in the setting of severe bacterial infection, particularly pneumococcal infection [22]. Finally, a fourth very recent study described associations between MBL level and polymorphisms and respiratory tract infections in young men [23]. These mixed and in some cases conflicting results reflect the variable disease phenotypes studied, encompassing non-invasive lower respiratory tract infection and invasive infection with a variety of bacterial species. As in our report, the number of individuals included in analysis of clinical outcome in these studies is often small and consequently associations with outcome should be interpreted with caution.

The lack of association between MBL deficiency and susceptibility specifically to pneumococcal empyema in our study may reflect the small size of this subgroup and hence a lack of power to detect such an effect. Previous genetic association studies of different infectious disease phenotypes have suggested that the effect of MBL is not limited to host defence against pneumococcus however, and indeed some *in vitro *experiments have demonstrated that MBL recognises and binds strongly to other Gram-positive bacteria. MBL deficiency might therefore be expected to predispose to the broader range of bacterial species that underlie empyema. Despite these results, our findings suggest that MBL does not play a major role in the development of thoracic empyema. The reported associations between MBL deficiency and poor outcomes in bacterial respiratory disease have raised the possibility that recombinant human MBL replacement therapy in deficient individuals may reduce such complications. Our data suggests that such an approach may not influence the development of thoracic empyema.

## Conclusions

MBL genotypic deficiency does not appear to significantly associate with susceptibility to thoracic empyema in humans. Larger studies are required to assess the possible effects of MBL deficiency on clinical outcome from empyema.

## Abbreviations

MBL: Mannose-binding lectin; MASP: MBL-associated serine protease.

## Competing interests

The authors declare that they have no competing interests.

## Authors' contributions

SJC performed the genotyping and analysis and wrote the article. NAM, CWHD, CEM, NPD and RJOD enrolled patients, collected samples and data, and defined phenotypes. RJOD and AVSH coordinated the study. SJC, FOV, CCK, AR, DWC, RJOD and AVSH contributed to the conception and design of the project. All authors critically revised the manuscript and approved the final article.

## Pre-publication history

The pre-publication history for this paper can be accessed here:

http://www.biomedcentral.com/1471-2350/11/5/prepub
